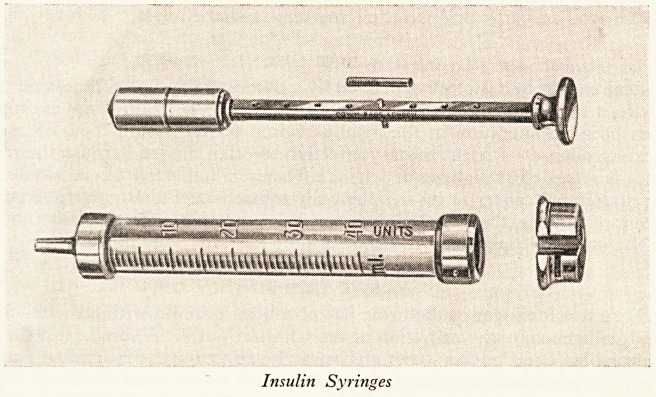# Home Care of the Diabetic

**Published:** 1955-11

**Authors:** C. T. Andrews

**Affiliations:** Consulting Physician, West Cornwall Clinical Area


### HOME CARE OF THE DIABETIC

BY

C. T. ANDREWS, M.D., F.R.C.P.

*Consulting Physician, West Cornwall Clinical Area*

Of all the chronic ailments affecting large numbers of the community diabetes is probably consistent with the highest degree of productivity. In most jobs the diabetic can compete on level terms with his fellow worker. If, however, this happy result is to be achieved for the maximum number there must be co-operation between the patient, his doctor and the hospital clinic.

This paper is not concerned with the initial problem of stabilization or with the treatment of major complications such as coma but with the day-to-day problems which arise in the lives of all diabetics. The initial stage of treatment and education will in the great majority of cases in this country have been undertaken by a medical or diabetic unit of the hospital service. Subsequently, however, the patient returns home to face the problems of school, work, or in the case of those not fit for an active job, to a varying measure of dependence in the home. The hospital team will only have done its job well if it has kept in mind the environment in which the patient lives and moves at home, the nature of his work and the arrangements for his meals. More vital perhaps than these is the patient's understanding of his disease and his willingness to co-operate. Time spent in enlisting the co-operation of the family is never wasted. It is more important perhaps that the wife who cooks the dinner should understand the dietetic arrangements better than her diabetic husband should.

### TRAINING THE PATIENT TO BE INDEPENDENT

It is usual before the patient leaves hospital to arrange for subsequent periodic attendance at a diabetic or general out-patient clinic. Dependence on the clinic however should not be encouraged, and one attendance every three months is probably sufficient for the average diabetic. From the outset the emphasis should be on normality. The diabetic is returning to lead a normal life. In all but exceptional cases he should be able to do anything which is reasonable for his age. The handicaps of diet and insulin should be regarded as necessary chores like having a bath or a shave or applying cosmetics. For the same reason the diabetic should not, in the absence of serious complications, be registered as a disabled person and should not be encouraged to carry sugar lumps to school or work. If stabilization has been efficiently done and the régime is being observed there is no need to remind the patient of the risk of hypoglycaemia.

All this is not to suggest that the régime taught in hospital is of slight importance. We now know that the well-controlled diabetic remains free of symptoms and of the more disabling complications for longer than the poorly controlled. It is also probable that he lives longer though this is perhaps a secondary consideration.

The secret of success lies in a regular routine, a thorough knowledge of the dietetic arrangements and the avoidance, as far as possible, of excesses and festivities. Temperament, education and family background therefore influence the prognosis in marked degree. I do not propose to describe the routine or dietetic arrangements, for these will vary from one patient to another and from one clinic to another. There are, however, certain mistakes and misunderstandings which are commonly seen by all those who have care of diabetics. It may be worth while to look at some of these.

### MISTAKES TO AVOID

Most diabetics seem quite incapable of understanding the significance of a unit of insulin. A typical interview in any diabetic clinic will illustrate this.

*Physician:* " Let me see Mrs. Brown what is your present dose of insulin?"

*Mrs. Brown:* " Twenty units from the blue and pink packet."

By which one is meant to understand that Mrs. Brown is having forty units of protamine zinc insulin. The commonest mistake arises through the substitution of a double- for a single-strength packet or vice versa. With the advent of a number of new insulins the colour scheme is becoming complicated but it should be possible for every diabetic to understand that a yellow packet always means single-strength (20 units to 1 c.c.), a blue packet double-strength (40 units to 1 c.c.) and a green packet quadruple-strength (80 units per c.c.). It is, however, a good plan when prescribing a fresh supply to get the patient to take the old packet to the chemist and insist that the new shall follow the same colour scheme. It is not unusual also for instructions from the clinic to the doctor to be similarly misinterpreted, twenty units being translated as forty because a double-strength packet was in use.

### INSULIN REACTIONS

These may be local or general. The local reactions are of two types. The first consists of a painful erythematous swelling at the site of injection. It is not due as is often suspected to infected syringes or needles and can usually be avoided by substituting one of the new "Lente" insulins for ordinary soluble or protamine zinc. The second consists of irregular areas of wasting at the site of injection. This is due to atrophy of the fatty tissues or insulin lipodystrophy. It does not respond to change in the type of insulin used and the best advice that can be given is that in giving injections areas should be avoided where the resulting deformity would be readily seen and that, as far as possible, injections should be given into the same sites. General insulin reactions are usually easily diagnosed. Apprehension, tremor, sweating, nervousness or coma of sudden onset occurring in the night or early morning where protamine zinc insulin is in use or before a main meal where soluble is used or after a spell of severe exertion or absention from diet gives the diagnosis readily. Confusion may arise, however, where a specimen of urine taken later in the day shows glycosuria or a specimen of blood reveals a high blood-sugar. It is not sufficiently realized that the blood-sugar in some diabetics may swing over a wide range in twenty-four hours. Thus an insulin-sensitive diabetic may exhibit symptoms of hypoglycaemia three hours after an injection of soluble insulin with a blood-sugar reading of 40 mg. per cent., and the same evening may be found to have a blood-sugar of 300 mg. with glycosuria and even ketosis.

It is a good general rule that attacks of hypoglycaemia which are not explicable by carelessness in diet or mistakes in insulin dosage indicate the need for reconsideration of the régime. A redistribution of the diet or of the daily insulin may be needed or a change to one or more of the Lente preparations.

### SIMPLE DOSES FOR THE AGED

In dealing with the aged or other group in which mistakes are likely to arise it is well to avoid complicated insulin dosage and also, if possible, the use of two insulins. I try to prescribe so far as possible in multiples of ten and to use protamine zinc, Lente insulin or failing these two daily and equal doses of Soluble.

### SPECIAL SYRINGE

In the administration of insulin dependence on the district nurse or other outside agency is to be avoided if at all possible. The nurse has many calls on her time and it is rarely possible to ensure absolute regularity in administration which is one of the secrets of success. It should indeed be the aim of the hospital staff to ensure that the patient so far as his insulin administration is concerned is quite independent not only of outside agencies but also of help in the home. From this point of view the intelligent schoolchild makes the best patient the older patient with cerebro-vascular degeneration or defective eyesight the worst. For the latter group and also for the younger patient with sub-normal intelligence the syringe illustrated on this page has been found useful. By means of a small adjustable peg on the piston shaft the capacity of the

*Insulin Syringes*

syringe can be regulated to take no more than the required dose. The syringe is most useful when one dose of insulin is required, e.g. twenty protamine zinc in the morning or two equal doses morning and afternoon or morning and evening.

### ASEPSIS AND BROKEN NEEDLES

Little need be said about the care of syringes for, though this may be a subject of endless discussion in fact sepsis following an injection of insulin is quite an uncommon complication. The time-honoured method of storing the syringe in spirit and rinsing it through with boiled water before use serves very well provided the spirit is changed once a week. A greater risk lies in the use of the fine hypodermic needle which breaks leaving a small piece deep in the subcutaneous tissues. If this should happen it is well to refer the patient to the casualty department of the nearest general hospital unless one end of the embedded portion is clearly superficial. The accident may be avoided by remembering to prescribe nothing smaller for home use than a number seventeen needle.

### DIETS

In arranging diet regard should also be had to the mental calibre of the diabetic. For the younger and more intelligent group the inculcation of the details of strict control by the Line Ration or similar scheme will give the best long-term results. For the less severe diabetic, for those of poor intelligence or advanced years it is better to rely on a simple qualitative diet sheet which shows lists of foods allowed, foods forbidden and foods permitted in specified quantities. A tendency to carelessness over the details of diet is fairly general whereas the fussy diabetic is comparatively rare.

### THE CAUSES OF OBESITY

Obesity constitutes a much commoner problem than weight loss particularly in patients having insulin. It is, of course, the rule for the patient who has started insulin therapy to gain weight. At what stage is weight gain to be regarded as a problem requiring more strict dietetic control? The answer to this question is generally to be found in a consideration of the patient's normal weight, i.e. that before the onset of the diabetes. When this is appreciably exceeded the curtailment particularly of carbohydrate intake becomes necessary usually with a corresponding reduction in insulin. Apart from rank disregard of the dietetic arrangements one of the commonest causes of substantial gain in weight is to be found in the habit which many diabetics acquire of treating proprietary diabetic bread, biscuits and jams as of no food value. This practice seems to be fostered by energetic salesmen. Obesity is best controlled by adjustment of diet and insulin alone and the use of amphetamine sulphate should if possible be avoided particularly in the hypertensive diabetic.

### WEIGHT LOSS

Persistent weight loss is often explained by a domestic crisis. An illness on the part of the wife, a bereavement or a period of financial anxiety. If these are excluded and there has been no alteration in the insulin rêgime search should then be made for complicating disease—a latent urinary infection so often missed because the resulting frequency is regarded as diabetic in origin, a dyspepsia indicative of gall stones, peptic ulcer or abdominal cancer or an incipient thyrotoxicosis. Pulmonary tuberculosis so often mentioned in textbooks is, in my experience, no commoner in diabetics than in any other comparable section of the population.

### URINE TESTING

Benedict's test for sugar in the urine has now been brought within the reach of the meanest intelligence by the provision of the Clinitest outfit. A similar test for ketosis is also available. The significance of glycosuria however for the individual patient requires careful consideration. In some patients with a low renal threshold for sugar repeated attacks of hypoglycaemia will often occur if negative tests throughout the twenty-four hours are demanded. In others where the renal threshold is high a fairly severe diabetic condition with troublesome complications such as retinitis may be masked by the complete absence of glycosuria. Persistent ketosis, however, must always be seriously regarded though again its significance varies. Some never exhibit it, others, particularly the insulin-sensitive diabetic, find it difficult to avoid.

### ADEQUATE CONTROL

In assessing progress it is well not to pay a too slavish regard to one particular feature, e.g. urine tests, but to consider the case as a whole. Ideally the well-controlled diabetic should be free of symptoms and complications and his urine should be persistently free of sugar and ketones. His blood-sugar should not exceed 200 mg. per cent. and his weight should not vary more than a few pounds from one month to another. In practice few attain this ideal. One may find persistent glycosuria with blood-sugars within the normal range or high blood-sugars with intermittent glycosuria and tendency to hypoglycaemia at particular times of the day. In general it may be said that the first consideration is to maintain the diabetic in good health and free of symptoms, that blood-sugar control is more important than freedom from glycosuria and that high blood-sugars at one time of the day with symptoms of hypoglycaemia at another generally indicate the need for a reconsideration of the insulin rêgime.

### DANGER OF MINOR ILLNESSES

Minor illness constitutes a considerable hazard particularly in the diabetic who is prone to ketosis. Major illness, e.g. haematemesis, generally brings the patient to hospital but the feverish cold with anorexia, the bout of infective diarrhoea, the urinary infection which is so common a complication in the female diabetic or the skin infection in the shape of a boil may readily give rise to a vicious circle in which the toxaemia aggravates the diabetes and the deteriorating diabetic condition interfers with control of infection. At the best an avoidable period of incapacity results: at the worst coma or a prolonged pyrexial illness may necessitate the patient's admission to hospital. Preventive measures at the outset will in most cases enable the infection to be contained and the vicious circle avoided.

The necessity for a high fluid and salt intake during such episodes is not fully realized. The diabetic at the onset of any feverish state should increase his fluid intake to six pints a day and, should ensure a steady intake of sodium chloride with all his meals, or if none are taken, in his drinks. Prompt use of the appropriate antibiotic, e.g. in urinary infection is most important, though this is not to encourage the indiscriminate use of penicillin for every bout of fever. In no circumstances should insulin be stopped merely because the patient has ceased to take his normal diet. Sweetened drinks should immediately be substituted for the carbohydrate which is omitted. If vomiting persists particularly with increasing ketosis intravenous glucose with saline becomes urgently necessary. At this stage hospital admission is generally desirable.

### AVOIDING FOOT COMPLICATIONS

Care of the feet constitutes a most important section of diabetic management. Peripheral gangrene starts here in more than 95 per cent. of cases and in a large proportion of these the initial injury which starts the gangrene is caused either by an ingrowing toe-nail or an abrasion from an ill-fitting shoe or an amateur attempt to pare a corn. The arteriosclerotic patient in whom the pulse cannot be felt in the dorsalis pedis or posterior tibial artery is particularly liable to this complication. It is a good habit to enquire about foot comfort every time one sees a diabetic patient. When an abrasion appears it is important to treat it promptly with a régime consisting of complete rest with elevation of the limb, dry dressings with a liberal use of spirit and powder and strict control by insulin. The gravest and commonest mistake made lies in the use of hot fomentations for these lesions. A fully developed ulcer will, of course, demand more elaborate measures. Foot complications are a common cause of prolonged incapacity and their prevention should therefore be continually in mind in dealing with these patients. The feet should be kept warm and dry, the socks should not be tight and, where the feet tend to perspire a talc dusting powder should be used each morning. The aged diabetic particularly should not pare his own corns but should leave this operation to the chiropodist or failing this to the district nurse. There are well-described effective measures of dealing with the ingrowing toe-nail. These should not wait till the presence of a serous exudate indicates that an ulcer has formed.

### SCHOOL AND OCCUPATION

A question frequently asked concerns the restriction of activity imposed by the disease in childhood. These questions frequently relate to secondary and university education, hard manual labour, school games, marriage and the bearing of children. While there are exceptions it can be said that in general diabetes is no bar to a higher education or to a professional career. Dietetic difficulties may arise where residence at a boarding-school or hostel is necessary but many schools and universities are now willing to co-operate in providing for this type of student. Strenuous games are best avoided owing to the risk of hypoglycaemia but there is generally no good reason to advise against manual work such as is involved in farming.

### MARRIAGE AND PREGNANCY

Advice may be sought particularly from the family doctor on the questions of marriage and childbearing. Some problems in this field still await solution but in general it may be said that there is little evidence that the well-controlled diabetic man or woman is less fertile than the normal individual of similar age, and that there is probably no increased risk of abortion though the risk of foetal death in the last weeks of pregnancy is considerable. This hazard has, however, been considerably diminished by the more careful control of the diabetes by insulin and by the modern practice of terminating the pregnancy by Caesarean section at the thirty-sixth week. The main risk to the foetus arises from the sudden development of ketosis in the mother at this stage and in the tendency to large babies which leads to obstetrical difficulties if the pregnancy is allowed to go to full term. The strictest supervision of the diabetes is necessary during the last few months of pregnancy and the closest possible co-operation between clinic and family doctor. Even in the best hands however, the foetal mortality is still about 20 per cent. The maternal mortality for the country as a whole is approximately 1 per cent. of diabetic pregnancies. It is still too early to assess the incidence of diabetes in the offspring of well-controlled diabetic parents but there is some indication that this may be high.

### CONCLUSION

The supervised and co-operative diabetic patient seldom finds himself in serious trouble. When he does the hospital service should give him first priority for his condition can seldom afford the long delay inherent in in-patient waiting lists today.

## Figures and Tables

**Figure f1:**